# Lightweight ECC-Based Self-Healing Federated Learning Framework for Secure IIoT Networks

**DOI:** 10.3390/s25226867

**Published:** 2025-11-10

**Authors:** Mikail Mohammed Salim, Farheen Naaz, Kwonhue Choi

**Affiliations:** 1 School of Computer Science and Engineering, Yeungnam University, Gyeongsan 38541, Republic of Korea; mikail@yu.ac.kr (M.M.S.); farheenaaz@yu.ac.kr (F.N.); 2 Department of Information and Communication Engineering, Yeungnam University, Gyeongsan 38541, Republic of Korea

**Keywords:** federated learning, blockchain security, elliptic curve cryptography, IoT security, privacy-preserving authentication, self-healing system, network security

## Abstract

The integration of federated learning into Industrial Internet of Things (IIoT) networks enables collaborative intelligence but also exposes systems to identity spoofing, model poisoning, and malicious update injection. This paper presents Leash-FL, a lightweight self-healing framework that combines certificateless elliptic curve cryptography with blockchain to enhance resilience in resource-constrained IoT environments. Certificateless ECC with pseudonym rotation enables efficient millisecond-scale authentication with minimal metadata, supporting secure and unlinkable participation. A similarity-governed screening mechanism filters poisoned and free-rider updates, while blockchain-backed checkpoint rollback ensures rapid recovery without service interruption. Experiments on intrusion detection, anomaly detection, and vision datasets show that Leash-FL sustains over 85 percent accuracy with 50 percent malicious clients, reduces backdoor success rates to under 5 percent within four recovery rounds, and restores accuracy up to three times faster than anomaly-screening baselines. The blockchain layer achieves low-latency consensus, high throughput, and modest ledger growth, significantly outperforming Ethereum-based systems. Membership changes are efficiently managed with sub-50 ms join and leave operations and re-admission within 60 ms, while guaranteeing forward and backward secrecy. Leash-FL delivers a cryptography-driven approach that unifies lightweight authentication, blockchain auditability, and self-healing recovery into a secure, resilient, and scalable federated learning solution for next-generation IIoT networks.

## 1. Introduction

The widespread adoption of the Internet of Things (IoT) has transformed critical domains such as healthcare, transportation, energy, and smart cities into highly data-driven environments. Billions of resource-constrained devices now continuously generate sensitive information, which, if efficiently harnessed, can enable predictive intelligence and autonomous decision-making [1]. Federated Learning (FL) has emerged as a compelling paradigm for such environments, allowing IoT devices to collaboratively train global models without directly sharing raw data, thereby preserving privacy and reducing communication overhead [2,3]. Despite these advantages, practical deployment of FL in IoT networks remains highly vulnerable to adversarial manipulation. Malicious clients may spoof identities, inject poisoned updates, perform free-rider attacks, or collude to degrade global model performance [4,5,6]. Left unaddressed, these vulnerabilities threaten both the reliability of trained models and the resilience of the underlying IoT infrastructure.

A range of techniques has been proposed to mitigate adversarial threats in federated learning. Lightweight cryptography, such as elliptic curve cryptography (ECC) and certificateless signatures, supports device authentication and key management [7,8]. Blockchain-enhanced FL frameworks introduce tamper-proof audit logs, decentralized trust, and tokenized incentives to improve accountability [9,10,11]. Robust aggregation methods—including clustering, similarity-based filters, and open-set recognition—help defend against poisoned or anomalous updates [12,13,14], while privacy-preserving approaches based on homomorphic encryption and trusted execution environments ensure data confidentiality during aggregation [15,16]. Each offers partial protection but also exhibits limitations: single trust anchors, high overhead, hardware or economic dependence, and no autonomous recovery once compromise occurs.

This fragmented landscape has fueled differing hypotheses. Some works claim that blockchain and incentives alone secure FL participation [17,18]; others stress cryptography or trusted hardware as decisive [19,20]; and still others rely solely on robust aggregation [21,22]. Yet recent studies show that none individually stop persistent poisoning or Sybil attacks once adversaries infiltrate the federation [23,24]. For example, blockchain-based intrusion detection in vehicular edge computing improves accountability but collapses under single-point aggregation failure, while open-set FL for zero-day detection enhances adaptability but lacks rollback for compromised rounds. These findings highlight the need for an integrated, lightweight framework that can both prevent and autonomously recover from adversarial threats in real-time IoT environments.

In Industrial IoT environments, the heterogeneity and limited capacity of edge devices make heavy cryptographic or incentive-based mechanisms impractical. A lightweight yet resilient security framework is therefore essential to protect federated learning without overwhelming constrained nodes. Elliptic curve cryptography offers strong authentication and key management with minimal computational cost, while blockchain provides decentralized auditability to ensure trust and accountability. By integrating these with a self-healing federated learning process, it becomes possible to achieve both security and continuity even under adversarial conditions.

Beyond lightweight security, Industrial IoT systems also demand continuous operational resilience. In these environments, model corruption or poisoning can occur unpredictably, and manual intervention to repair the federated model is often impractical due to real-time constraints. To address this, self-healing federated learning enables the system to automatically detect, isolate, and recover from compromised or low-integrity updates through rollback and reconfiguration mechanisms. This autonomous recovery ensures that learning remains stable and trustworthy even under persistent or evolving adversarial conditions, thereby maintaining uninterrupted operation across critical IIoT networks.

In addition to ensuring lightweight authentication, achieving trustworthy coordination among distributed IIoT participants requires a verifiable and tamper-proof record of model updates. Blockchain serves this purpose by providing decentralized consensus, immutable logging, and transparent auditability of federated transactions. It removes reliance on a single central aggregator and allows participants to validate updates collectively through smart contracts. This decentralized audit layer not only strengthens accountability but also supports the proposed self-healing mechanism by enabling secure checkpoint recovery and traceable rollback to verified states.

To address these challenges, this paper proposes Leash-FL, a Lightweight ECC-Based Self-Healing Federated Learning framework for Secure IoT Systems. Leash-FL combines certificateless ECC-based pseudonym authentication with blockchain-audited membership management and similarity-governed edge screening to filter poisoned or low-effort updates. At the cloud layer, a blockchain-assisted self-healing controller monitors anomalies and executes rollback to signed checkpoints, reconfigures screening thresholds, and refreshes pseudonyms and group keys, thereby ensuring continuous and trustworthy global model updates. By design, Leash-FL eliminates fragile trust anchors, avoids excessive cryptographic or hardware overhead, and ensures recovery from poisoning or Sybil attacks without service interruption.

The main contributions of this study are threefold:

- Lightweight authentication and unlinkability: We design an ECC-based certificateless pseudonym scheme with dynamic rotation and blockchain-anchored accountability, removing the need for centralized trust.
- Edge-level robustness with auditable screening: We introduce similarity-governed pre-aggregation filters combined with blockchain audit trails to down-weight or reject anomalous contributions, ensuring scalable and transparent validation.
- Blockchain-assisted self-healing: We propose a checkpoint-driven rollback and reconfiguration mechanism that autonomously recovers from adversarial rounds, enforces forward/backward secrecy, and maintains uninterrupted service.

Extensive experimental validation demonstrates that Leash-FL sustains model accuracy under poisoning, Sybil, and free-rider attacks while incurring minimal computational and communication overhead compared to existing approaches. These results establish Leash-FL as a resilient and lightweight framework capable of securing federated learning in heterogeneous IoT networks.

## 2. Related Works

Recent research on blockchain-enabled federated learning (FL) has focused on enhancing security, privacy, and trust across IoT and edge environments. The main research trends can be grouped into four directions: lightweight authentication, blockchain-based audit and incentive mechanisms, privacy-preserving aggregation, and domain-specific intrusion detection frameworks. Ten representative studies are reviewed below in relation to the proposed Leash-FL framework.

TrustBCFL [25] integrates blockchain-assisted update validation and reputation mechanisms to mitigate bias in FL aggregation for IoT systems. It achieved a 4–7% accuracy improvement on IoT anomaly datasets compared with vanilla FL but remains vulnerable to poisoning when malicious updates bypass the bias filter. Leash-FL extends auditability with checkpoint-based rollback and self-healing to prevent persistent corruption. BIT-FL [26] introduces token-based incentives, rewarding honest participants and penalizing malicious ones through blockchain and elliptic curve signatures. It attained 92–94% accuracy on CIFAR-10 and MNIST under up to 30% backdoor attacks. However, economic deterrence cannot prevent long-term adversaries. Leash-FL enforces resilience cryptographically through certificateless ECC and similarity screening.

VEH-FL [27] applies blockchain to vehicular edge computing, where roadside units (RSUs) aggregate intrusion-detection models. Although detection accuracy improved over FedAvg, the RSU-centric setup creates bottlenecks and single points of failure. Leash-FL distributes validation across edge cohorts and uses blockchain logs to remove such dependencies. Zero-X [28] employs deep autoencoders and a Proof-of-Accuracy consensus to detect zero-day attacks, achieving >95% accuracy on N-day and >85% on 0-day datasets under non-IID distributions. Once poisoned models bypass validators, however, Zero-X cannot recover. Leash-FL introduces checkpointed rollback and CRT-based rekeying to reverse compromise without halting training.

IDFLM-ES [29] combines a deep belief network with Golden Jackal and Dung Beetle Optimization, reaching 98.24% accuracy on Edge-IIoT while reducing training time versus CNN and RNN baselines. Yet its hybrid design is computation-heavy for constrained IoT devices. Leash-FL achieves comparable resilience using lightweight ECC authentication and edge-side similarity filtering. TEE-FL [30] secures aggregation via blockchain and trusted execution environments (SGX), encrypting local updates within enclaves. Accuracy on MNIST, Fashion-MNIST, and CIFAR-10 dropped only 2–3% from baseline FL, but SGX introduces side-channel and rollback vulnerabilities plus scalability limits. Leash-FL achieves software-level integrity without hardware trust anchors.

OpenFL [31] enables permissionless FL through Ethereum staking, rewarding valid contributions. Accuracy exceeded 95% on MNIST and 82–85% on CIFAR-10, yet gas fees (≈0.01–0.02 ETH/round) and latency hinder IoT use. Leash-FL confines blockchain use to lightweight audit proofs, remaining cost-efficient. PBFL [32] adopts Paillier homomorphic encryption for privacy-preserving FL, reducing communication overhead by 30% and maintaining accuracy within 1–2% of FedAvg on MNIST and CIFAR-10. However, per-round latency rose 1.8× and no rollback exists once poisoned models are aggregated. Leash-FL employs ECC signatures and checkpointed recovery to address both efficiency and resilience.

IoT-BFLA-ML [33] utilizes a blockchain-enhanced Gaussian Bayesian Transfer CNN for consumer IoT, achieving 91% accuracy and latency reduction of 71% on LFW, CelebA, and CASIA-WebFace datasets. Its reliance on heavy CNNs and centralized aggregation restricts scalability. Leash-FL uses lightweight ECC authentication and distributed validation suited to resource-constrained environments. BFLIDS [34] integrates smart contracts, IPFS storage, and MongoDB to secure collaborative intrusion detection for IoMT networks. High F1-scores were achieved on Edge-IIoTset and TON-IoT datasets, but contract execution and off-chain storage add latency and scalability concerns. Leash-FL avoids contract overheads by combining blockchain-audited screening with lightweight ECC authentication and self-healing re-admission.

Overall, current frameworks rely on single trust anchors [25], token incentives [26,31], hardware enclaves [30], computationally heavy deep models [29,33], or costly encryption [32]. Most also lack rollback or membership renewal [28,32] and face scalability issues from smart contract overhead [34]. Leash-FL overcomes these gaps by integrating certificateless ECC-based authentication, blockchain-anchored similarity screening, and checkpoint-driven self-healing into a unified, efficient architecture for secure FL in IIoT environments. A summary of the comparison of blockchain-enabled federated learning frameworks and Leash-FL is provided in Appendix A.

## 3. Proposed LEASH-FL Framework

In the considered IIoT environment, the federated learning system is exposed to multiple categories of adversaries with varying objectives and capabilities. (1) Poisoning adversaries aim to manipulate the global model by injecting falsified or low-quality updates, thereby degrading learning accuracy or biasing model outcomes. (2) Sybil or impersonation attackers attempt to create multiple fake identities or reuse compromised credentials to distort the aggregation process or flood the federation with malicious nodes. (3) Free-rider participants try to obtain model benefits without contributing meaningful updates, exploiting trust among peers. In addition, eavesdropping and inference adversaries may seek to reconstruct private client data through gradient leakage or side-channel analysis. Together, these behaviors represent the key threats that motivate the design of Leash-FL’s certificateless authentication, blockchain auditability, and self-healing mechanisms to ensure confidentiality, integrity, and resilient operation in distributed IIoT environments.

This paper aims to present a lightweight ECC-based self-healing federated learning framework for secure IoT systems, termed Leash-FL. Modern IoT deployments remain vulnerable to identity spoofing, poisoned or free-rider updates, and Sybil attacks, while most defenses lack autonomous recovery. Leash-FL addresses these challenges by combining certificateless ECC-based authentication with similarity-governed pre-aggregation screening and a blockchain-assisted self-healing controller that can roll back and reconfigure training without service interruption.

### 3.1. Overview of the Proposed LEASH-FL Framework

**Figure 1:**
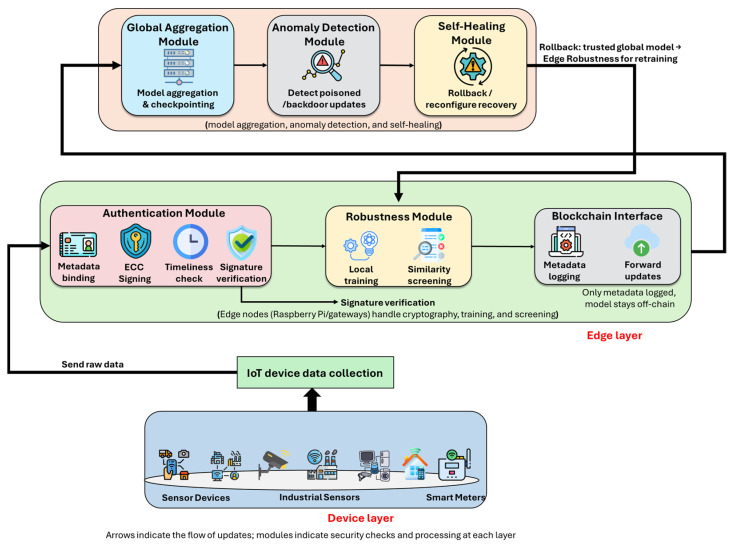
Overview of the Leash-FL framework.

Figure 1 illustrates the overall architecture of the proposed Leash-FL framework, which operates across three collaborative layers: device, edge, and cloud.

At the device layer, heterogeneous IoT endpoints (sensors, wearables, meters, and vehicular nodes) locally train models on private data and transmit only signed model updates authenticated through pseudonymized certificateless ECC credentials. Each update is timestamped to ensure freshness and accompanied by lightweight training-effort metadata. Raw features and labels never leave the device, preserving data privacy and unlinkability across rounds.

The edge layer hosts gateway nodes that act as local coordinators. They verify signatures and timestamps, execute similarity-governed screening to filter low-effort or anomalous submissions, and record audit proofs (hashes, signatures, timestamps, and verdicts) on a lightweight consortium blockchain for tamper-evident auditing and membership governance. Only verified updates are forwarded to the cloud for aggregation.

The cloud layer performs weighted aggregation of validated updates to produce the global model and maintains signed checkpoints to enable rollback and self-healing when anomalies are detected. It coordinates revocation and reconfiguration through blockchain-backed control, refreshing pseudonyms and group keys for affected devices without exposing sensitive model parameters.

Together, these layers establish a continuous, privacy-preserving, and tamper-resistant federated learning process that supports dynamic IoT environments. They collectively enable system initialization, authenticated training, edge-side verification, blockchain-based audit, global aggregation, self-healing, redistribution, and adaptive membership management. The detailed operational flow—corresponding to these nine sequential stages—is described in Section 3.1.1, Section 3.1.2, Section 3.1.3, Section 3.1.4, Section 3.1.5, Section 3.1.6, Section 3.1.7, Section 3.1.8 and Section 3.1.9.

#### 3.1.1. System Initialization and Credential Management

The initialization and credential management module forms the cryptographic foundation of Leash-FL, ensuring that only authorized IoT devices can participate in federated learning while maintaining privacy, unlinkability, and scalable key management. It encompasses four main components: system setup, pseudonym generation, certificateless key establishment, and dynamic membership management.

During **system setup**, a trusted authority (TA) defines an elliptic-curve group G of order q with base point P, chooses a master secret $s\in Z_{q}^{*}$, and computes the public key

(1) $$P_{pub}=s.P$$

which serves as the root of trust for all participants. The TA also defines several cryptographic hash functions $H_{0},H_{1},H_{2},H_{3}$ used for pseudonym binding and key derivation. These parameters $\left\{q,P,G,P_{\mathrm{pub}},H_{0},H_{1},H_{2},H_{3}\right\}$ are publicly distributed, while the master key s remains secret.

In the pseudonym generation stage, each device $w_{i}$ with identity $ID_{i}$ receives a pseudonym $PID_{i}$ that conceals its real identity while maintaining accountability. The pseudonym is computed through a hash-binding process that links $ID_{i}$ with a random seed and a time validity token. This ensures that even if updates from the same device appear in consecutive rounds, their pseudonyms remain unlinkable. When the validity window expires.

Certificateless key establishment eliminates the need for traditional public-key certificates. The TA generates a partial private key for each device, while the device independently produces its own secret and combines the two to form a complete signing key pair. The hash of the pseudonym and public parameters acts as a linking factor, enabling any verifier to authenticate the signature without relying on a certificate chain. This dual-source key generation prevents key escrow and protects against impersonation by ensuring that neither the TA nor the device alone possesses the complete key. The specific mathematical expressions for the key generation and verification process are provided in Appendix B.1 (Equations (A1) and (A2)).

Dynamic membership management handles secure joining, leaving, and rekeying of devices in the network without interrupting training. Leash-FL employs a Chinese Remainder Theorem (CRT)–based rekeying protocol in which each participant is assigned a unique modulus, and all moduli are pairwise co-prime. When a member joins or leaves, the cloud generates a composite modulus and distributes a new rekey parameter that allows only active members to reconstruct the updated key while excluding revoked ones. The group-key consistency condition is expressed as

(2) $$a^{\prime }modb_{i}=k_{d}^{\prime },\forall i\in \{1,\ldots ,n\},i\neq r$$

where r denotes the revoked node. The full CRT derivation and its correctness proof appear in Appendix B.1 (Equation (A3)). This rekeying design guarantees both forward secrecy (revoked nodes cannot compute future keys) and backward secrecy (new nodes cannot access past models).

To maintain transparency, all membership updates, key-refresh events, and revocation records are anchored on a lightweight blockchain maintained at the edge. Each transaction logs the pseudonym, event type, and validity period of the rekeyed group secret, forming an immutable record of system reconfiguration.

Through this integrated ECC–CRT initialization, Leash-FL achieves secure pseudonymized participation, certificate-free key generation, and privacy-preserving rekeying. This foundation ensures that all subsequent phases—training, verification, and aggregation—operate within a trustworthy cryptographic domain, balancing efficiency with verifiable security.

#### 3.1.2. Local Training and Authentication

Once initialization and credential management are completed, each IoT device participates in federated learning by training on its local dataset and producing authenticated model updates for aggregation. This module guarantees that raw data always remains on the device, while only signed parameter updates and lightweight metadata are transmitted for validation.

At the beginning of round t, each device $w_{i}$ receives the authenticated global model parameters $W_{g}^{t-1}$ from the cloud through the edge coordinator and updates them locally using stochastic gradient descent (SGD). After several epochs, the new model parameters are:

(3) $$W_{g}^{t}\leftarrow W_{g}^{t-1}-\frac{\eta }{\left|D_{i}\right|}\sum_{(X,Y)\in D_{i}}\nabla L\left(f\left(X,W_{g}^{t-1}\right),Y\right),$$

where η is the learning rate, $|D_{i}|$ is the local dataset size, and L(⋅) denotes the loss function. This expression defines the local update $W_{i}^{t}$ that is later shared with the federation.

After training, the device signs its update using its certificateless key pair. The signing process involves computing a hash-based authentication coefficient and a lightweight ECC signature derived from the current group key. The detailed mathematical signing and message-construction steps are listed in Appendix B.2 (Equations (A4)–(A6)). Each signed message includes the updated model $W_{i}^{t}$, the pseudonym $PID_{i}$, and a timestamp $T_{i}$ to ensure freshness.

The signature’s correctness is verified at the edge or cloud using:

(4) $${sgn}_{i}*P=U_{i}+h_{i2}(L_{i}+h_{i1}*P_{pub})$$

where $L_{i}$ and $h_{i1}$ originate from the certificateless key generation, and $P_{\mathrm{pub}}$ is the TA’s public key. This relation confirms the authenticity and integrity of each update and prevents forgery or impersonation. Here, “∗” denotes elliptic curve scalar multiplication.

The local training and signing phase enforces several security guarantees. Data privacy is preserved because raw features never leave the device. Authenticity and integrity are ensured through ECC-based signatures, while non-repudiation prevents a device from denying participation once its update is recorded. Training-effort metadata attached to each signed message deters free-rider attacks by making it infeasible to submit random or untrained updates. Including timestamps defends against replay attempts, allowing the edge layer to reject stale submissions. Collectively, these safeguards ensure that only valid, authenticated, and timely updates participate in aggregation, providing the foundation for secure and reliable model training in Leash-FL.

#### 3.1.3. Edge-Side Verification and Screening

Upon receipt of a signed message $M_{i}=\{W_{i}^{t},PID_{i},T_{i},(U_{i},sgn_{i})\}$, the edge coordinator first enforces timeliness. Messages are accepted only if their timestamps fall within the configured freshness window; otherwise, they are discarded as stale or replayed. This freshness check, consistent with the timing logic used for FL control packets, is performed before any cryptographic operation to minimize computation overhead.

For messages that pass freshness verification, the edge authenticates the sender using the certificateless signature relation

(5) $${sgn}_{i}*P=U_{i}+h_{i2}\left(L_{i}+h_{i1}*P_{pub}\right)+a_{i}K_{pub}$$

where $h_{i1}=H_{2}(PID_{i},P_{\mathrm{pub}},R_{i})$, $h_{i2}=H_{3}(W_{i}^{t},PID_{i},PK_{i},U_{i},T_{i})$, and $a_{i}=H_{1}(PID_{i},T_{i})$. This equality holds only if the update originates from a legitimate device possessing valid keys. Invalid or replayed messages are rejected immediately. When multiple updates arrive simultaneously, the edge performs **batch verification** to amortize elliptic-curve computations and sustain throughput. Additional details of this process are summarized in Appendix B.3 (Equations (A7) and (A8)).

After authentication, the edge performs **similarity-governed screening** to suppress low-effort or anomalous updates. For the round’s candidate set $C_{t}=\{W_{i}^{t}\}$, each update is compared to a robust reference ${\bar{W}}^{t}$ (e.g., the coordinate-wise median) using cosine similarity,

(6) $$s_{i}=\frac{vec\left(W_{i}^{t}\right)*vec({\bar{W}}^{t})}{\left|\left|vec\left(W_{i}^{t}\right)\right|\right|\left|\left|vec\left({\bar{W}}^{t}\right)\right|\right|}$$

Updates with $s_{i}<\tau_{s}$ are flagged as potential outliers. Magnitude checks then evaluate the update norm and discard any whose scale deviates beyond the configured thresholds. When a small validation buffer is available, the contribution quality is also estimated through local loss reduction, $\Delta L_{i}$; updates showing modest improvement are down-weighted rather than rejected. The resulting verdicts are classified as {accept,down–weight,reject} with associated weights $w_{i}$.

All screening outcomes are immutably recorded on a lightweight blockchain distributed across edge servers. Each record includes the hash of the submitted update, pseudonym, timestamp, and verdict, ensuring accountability and traceability while model tensors remain stored off-chain to preserve efficiency and confidentiality. Finally, the edge forwards the screened and weighted batch $\left\{(W_{i}^{t},w_{i})\right\}$ to the cloud, together with a reference to the blockchain transaction for traceability. This guarantees that only fresh, authenticated, and similarity-consistent updates participate in global aggregation while every step remains auditable.

#### 3.1.4. Blockchain-Based Audit and Membership Management

In Leash-FL, a permissioned blockchain deployed across edge servers maintains an immutable audit trail and decentralized membership control. After edge-side verification and screening, each round’s accepted or rejected updates are encapsulated as metadata records linked to the ledger. For a device $w_{i}$, the record includes the hash of its submitted update, pseudonym $PID_{i}$, timestamp $T_{i}$, and screening verdict $v_{i}\in \{accept,down\text{–}weight,reject\}$. The record hash is defined as

(7) $$R_{i}=H(W_{i}^{t}||{PID}_{i}||T_{i}||v_{i})$$

where H(⋅) denotes a collision-resistant hash function. These digests form candidate blocks for the round, and consensus is reached through validation votes among a committee of edge nodes. Each edge $E_{j}$ verifies the update Δ$W_{i}$ and casts a binary vote; the formal tally process is provided in Appendix B.4 (Equation (A9)). An update is committed once a majority of edges approve it.

To preserve ledger integrity, each block $B_{t}$ is linked to its predecessor through a cryptographic digest such that

(8) $$H(L_{t})\neq H(L_{t}^{\prime })$$

for any tampered log $L_{t}^{\prime }$. This condition ensures that unauthorized alterations in the audit trail are immediately detectable by hash mismatch across replicas.

**Membership management** is integrated into the same ledger. Each participant is assigned a CRT-based modulus and contribution that enables group-key updates during join or leave events. When a member is revoked, its contribution is removed and a new key is computed from the remaining set, while new devices contribute fresh CRT components to the sum. The detailed formulas for these operations are listed in Appendix B.4 (Equations (A10) and (A11)). This procedure guarantees forward secrecy (revoked devices cannot compute future keys) and backward secrecy (new devices cannot reconstruct previous ones).

By anchoring membership events and rekeying transactions on-chain, Leash-FL provides tamper-proof auditability and verifiable synchronization across edges. Only metadata and hash digests are stored on the ledger, keeping storage and communication overhead bounded while preserving transparency, accountability, and trust for all participants.

#### 3.1.5. Security Model and Proof Sketches

We define the security model of Leash-FL under standard assumptions in certificateless public-key cryptography and federated learning. The adversary A is modeled as a probabilistic polynomial-time (PPT) adversary capable of compromising up to a fraction m of clients (10–50%) and controlling their local training. It may request chosen-message signatures (CMA), replay stale updates, spawn multiple pseudonyms (Sybil), or read all blockchain data but cannot modify committed transactions because of consensus guarantees. The adversary cannot derive private keys unless it can solve the Elliptic Curve Discrete Logarithm Problem (ECDLP), which is assumed infeasible.

The security objectives of Leash-FL are fourfold. First, it ensures authenticity and integrity, meaning that only legitimate clients can produce and submit accepted model updates. Second, it guarantees unforgeability, preventing any adversary from generating valid signatures without the corresponding private keys. Third, it provides forward secrecy, ensuring that revoked clients cannot derive session keys or access future updates after being removed from the federation. Finally, it maintains backward secrecy, preventing newly joined clients from reconstructing or accessing past session keys and model parameters. All proofs are based on the correctness of the certificateless signature scheme and the CRT-based rekeying process described earlier (see Appendix B.5 for formal definitions).

**Lemma** **1.** *(Authenticity and Integrity)*

*Assumption. Adversary *A *has PPT capabilities and can intercept or modify updates but not forge ECC signatures without private keys.*
*Proof Sketch. Each local update includes a valid ECC signature bound to a pseudonym. Edge servers verify these signatures using public parameters before aggregation. Any modification causes signature mismatch and rejection with probability 1, ensuring authenticity and integrity.*

**Lemma** **2.** *(Unforgeability)*

*Assumption. Adversary *A *has access to chosen-message signing queries (CMA).*
*Proof Sketch. If *A *produces a valid forgery without the private key, it would imply the ability to solve the ECDLP in group *G *. Because ECDLP is infeasible, the probability of forgery is negligible. Hence, the certificateless ECC signature scheme in Leash-FL is existentially unforgeable under chosen-message attack (EUF-CMA).*

**Lemma** **3.** *(Forward Secrecy)*

*Assumption. Adversary *A *possesses all past keys of a compromised device but no access to ongoing rekeying transactions.*
*Proof Sketch. Forward secrecy follows from CRT-based rekeying. Upon revocation, new session keys are generated and broadcast through the blockchain ledger; pseudonyms and partial keys are refreshed. Old keys become obsolete, preventing revoked clients from deriving future ones.*

**Lemma** **4.** *(Backward Secrecy)*

*Assumption. Adversary *A *represents a revoked client holding its last valid key.*
*Proof Sketch. After revocation, the trusted authority issues a new group key distributed only to active participants through the ledger. Revoked clients are excluded from rekeying and cannot compute fresh session keys. Since pseudonyms and secrets are refreshed, backward secrecy holds.*

Under this model, Leash-FL achieves authenticity, integrity, unforgeability, and secrecy guarantees against polynomial-time adversaries. These properties are grounded in the hardness of ECDLP and the correctness of blockchain-driven rekeying.

#### 3.1.6. Global Aggregation and Checkpointing

Following edge-side verification and similarity screening, only authenticated and quality-checked updates are forwarded to the cloud. These validated contributions form the input for global aggregation, where weighted averaging and batch verification ensure both efficiency and robustness. If anomalies are detected during or after aggregation, corrective actions are triggered through the self-healing process described in Section 3.1.7, enabling rollback to trusted checkpoints or adaptive reconfiguration of the model. Together, these operations establish a secure end-to-end pipeline for federated learning in Leash-FL.

After receiving the screened and weighted updates $\left\{(W_{i}^{t},w_{i})\right\}$ from the edge servers, the cloud verifies each blockchain reference to confirm that the updates were correctly authenticated and screened. The global model is then updated using weighted averaging, expressed as

(9) $$W_{g}^{t}=\frac{\sum_{i=1}^{m}w_{i}*W_{i}^{t}}{\sum_{i=1}^{m}w_{i}}$$

where $w_{i}$ denotes the dataset size, similarity score, or penalty factor assigned by the edge layer. This ensures that contributions from well-behaved devices have greater influence, while unreliable or borderline updates have proportionally less impact.

To enhance efficiency, the cloud performs **batch signature verification** on multiple authenticated updates. Using a small-exponent method, all signatures are verified in a single computation step, significantly reducing elliptic-curve overhead. The detailed verification expression is provided in Appendix B.6 (Equation (A12)).

After aggregation, the newly generated global model is stored as a **checkpoint**. The controller maintains a fixed number K of recent checkpoints, forming a rolling window of trusted states. If future anomalies or adversarial behaviors are detected, the system can revert to the latest valid checkpoint rather than restarting training from scratch, thereby minimizing downtime and cost.

At the conclusion of each round, the checkpointing mechanism guarantees that at least one verified global model is always available for recovery. Even with robust screening at the edge and weighted aggregation at the cloud, rare adversarial updates or coordinated Sybil behaviors may still slip through. To handle such cases, Leash-FL employs a dedicated self-healing monitor that continuously evaluates the aggregated model and initiates rollback whenever anomalies are detected.

#### 3.1.7. Self-Healing Response

To maintain resilience against malicious or corrupted updates, the cloud continuously monitors the aggregated model for anomalies. Detection relies on indicators such as sudden drops in validation accuracy, a surge in rejected updates, or deviations in similarity scores. Formally, an anomaly is triggered when the cosine similarity between a local update and the cohort reference falls below the acceptance threshold α:

(10) $$\cos\left(\theta_{i}\right)=\frac{W_{i}^{t}*{\bar{W}}^{t}}{\left|\left|W_{i}^{t}\right|\right|\left|\left|{\bar{W}}^{t}\right|\right|}<\alpha$$

where ${\bar{W}}^{t}$ denotes the reference model. When an anomaly is detected, the cloud activates the **self-healing response** R(t), which either rolls the system back to a previously trusted checkpoint or performs adaptive reconfiguration of the model. The formal definition of R(t) is given in Appendix B.7 (Equation (A13)).

During operation, authenticated and screened updates are aggregated through weighted averaging and batch verification. If inconsistencies appear in later rounds, the self-healing process determines whether to restore the last verified model or to adaptively exclude outliers and recompute aggregation with stricter thresholds. Rollback reinstates the latest trusted checkpoint, while reconfiguration recalibrates similarity thresholds, regenerates pseudonyms, and refreshes group keys for the affected cohort.

Each self-healing event, rollback, pseudonym refresh, key rotation, or threshold update, is immutably recorded on the blockchain ledger, providing traceability without exposing model parameters. Through this synergy of weighted aggregation, anomaly detection, and checkpoint-based recovery, Leash-FL remains resilient to poisoning, Sybil, and free-rider attacks. At the same time, blockchain-anchored logging ensures that every recovery action is auditable and tamper-evident, preserving the trustworthiness of federated learning across all participants.

#### 3.1.8. Redistribution and Synchronization

After aggregation and self-healing, the updated global model $W_{g}^{t}$ must be reliably disseminated to all legitimate devices. The cloud broadcasts $\left\{W_{g}^{t},h_{m},C_{T_{t}},\sigma \right\}$, where $h_{m}=H(W_{g}^{t}||C_{T_{t}})$ is the hash digest for the current round timestamp $C_{T_{t}}$, and σ is the cloud’s certificateless ECC signature. Each device verifies both freshness and authenticity of the broadcast through,

(11) $$Verify({\sigma ,W}_{g}^{t},{CT}_{t})\wedge H(W_{g}^{t}||{CT}_{t})=h_{m}$$

which ensures that the received model originates from the legitimate cloud and has not been altered in transit.

To maintain synchronization, each device returns a short acknowledgment containing its pseudonym and the hash of the received model. The edge aggregates these acknowledgments and writes the resulting digest to the blockchain as a tamper-evident proof of round completion. The detailed expressions for the acknowledgment and late recovery verification appear in Appendix B.8 (Equations (A14) and (A15)).

Devices that fail to acknowledge within the round window are classified as **stragglers**. They can resynchronize by retrieving the last valid digest and receipt $\left\{h_{m},C_{T_{t}}\right\}$ from the blockchain and requesting the corresponding model from the edge. Since all model digests are stored on-chain, any device can independently verify consistency with the global state before resuming participation.

From a performance perspective, redistribution requires only a single signature verification and one hash operation per device, keeping overhead minimal. Edge aggregation of acknowledgments scales linearly with the number of participants, while blockchain logging grows proportionally to the number of rounds. As only metadata and hashes are stored on-chain, bandwidth costs for transmitting full model weights remain low.

By combining authenticated broadcast, acknowledgment-based synchronization, and blockchain-backed recovery, Leash-FL ensures that every participant trains on the same trusted global model. This integrated design provides verifiable resistance against replay, tampering, and equivocation attacks, ensuring synchronization and consistency across the federation.

#### 3.1.9. Dynamic Join/Leave with Re-Admission

The Leash-FL framework supports dynamic membership to accommodate the highly variable nature of IoT environments, where devices may join, leave, or require re-admission after quarantine. Membership events are coordinated through certificateless credentials, CRT-based rekeying, and blockchain-backed logging to ensure accountability, secrecy, and synchronization across training rounds.

When a new device $w_{j}$ is admitted, the trusted authority issues a fresh pseudonym $PID_{j}$ and a corresponding certificateless key pair. The controller integrates the new device into the federation by extending the CRT-based rekeying process previously described in Appendix B.4 (Equations (A10) and (A11)). These same relations govern both join and leave events: adding a new participant contributes a fresh CRT component to the global accumulator, while revocation or voluntary departure removes the corresponding term. As a result, active members derive an updated group key, newly admitted devices gain access only to the current key, and revoked devices lose the ability to compute future ones—preserving both forward and backward secrecy.

When a device misbehaves, its pseudonym and corresponding CRT component are excluded, and a new group key is distributed solely to active members. For repeated misconduct under rotating pseudonyms, the trusted authority can resolve the true identity from the pseudonym–timestamp mapping and permanently revoke the device from future participation.

All membership events—including joins, leaves, rekeying operations, and re-admission—are immutably recorded on the blockchain ledger. Each transaction logs the pseudonym, event type, validity period, and rekeying version, creating an auditable history of membership evolution without exposing private data. Devices placed in quarantine may later be re-admitted following successful re-attestation of credentials. Re-admission is treated as a fresh join, involving a new pseudonym issuance and CRT-based reintegration to prevent access to any previous session keys.

Through this integrated mechanism, dynamic membership management in Leash-FL achieves scalability, accountability, and robustness. Joins and leaves are processed efficiently using modular CRT arithmetic, while blockchain consensus provides tamper-proof traceability across membership churn. This ensures that federated learning remains secure and synchronized even in highly dynamic IoT environments characterized by device mobility, heterogeneity, and adversarial behavior.

In summary, Leash-FL provides a complete end-to-end framework for secure and resilient federated learning in IoT environments. It begins with certificateless credential initialization, followed by authenticated local training, edge-side verification, and blockchain-audited screening. Weighted aggregation, checkpointing, and self-healing preserve integrity and enable adaptive recovery from anomalies, while authenticated redistribution ensures model consistency across devices. Finally, CRT-based rekeying and blockchain-backed membership management maintain forward and backward secrecy during join, leave, and re-admission events. Collectively, these components enable scalable, trustworthy, and verifiable learning across heterogeneous IoT systems.

## 4. Evaluation Results

Before presenting the detailed experimental setup and results, it is useful to provide an overview of the benchmark design and evaluation objectives. The experimental evaluation of Leash-FL is organized to validate the framework’s overall efficiency, security, and scalability in Industrial IoT environments. The benchmarks span both constrained-device experiments (capturing cryptographic and communication overhead on IoT-class hardware) and federated learning benchmarks (covering intrusion, anomaly, and image-classification tasks). The evaluation further compares Leash-FL against representative blockchain-enabled FL frameworks such as PBFL, OpenFL, and Ethereum-FL to highlight relative gains in authentication latency, adversarial resilience, ledger efficiency, and membership management.

The evaluation of Leash-FL aims to demonstrate that the proposed framework achieves lightweight authentication, blockchain-anchored accountability, and dynamic membership management while preserving feasibility in heterogeneous IoT environments. To establish this claim, we systematically measure four dimensions: (i) the computational and communication cost of certificateless ECC authentication on IoT-class devices; (ii) blockchain auditability and consensus performance, with emphasis on metadata-only logging and low consensus latency; (iii) energy feasibility, validating that cryptographic overhead remains in the millijoule range relative to typical IoT battery capacity; and (iv) membership dynamics, ensuring forward and backward secrecy under client churn through revocation and rekeying. The evaluation design combines constrained-device benchmarks with blockchain network tests, enabling results to be directly compared with representative security frameworks such as PBFL and Ethereum-FL.

### 4.1. Experimental Setup

To validate the effectiveness of Leash-FL, experiments were conducted focusing on authentication efficiency, adversarial robustness, blockchain auditability, and membership management. The evaluation reflects realistic IoT deployments while maintaining comparability with standard federated learning benchmarks.

A diverse set of datasets was used for intrusion detection, anomaly detection, zero-day attack evaluation, and image classification. The Edge-IIoT, UNSW-NB15, CIC-IDS2017, Google Cluster, 5G-NIDD, and VDoS datasets provided network and workload traces for intrusion and anomaly detection, while MNIST, CIFAR-10, and FEMNIST served as lightweight benchmarks for privacy- and blockchain-FL baselines (Table 1). Corresponding model architectures—MLPs, CNNs, and ResNet-based variants—were configured for each domain as summarized in Table 2.

The parameter settings in Table 2 and Table 3 were selected to ensure both representativeness and fairness in evaluating Leash-FL across heterogeneous IoT environments. Lightweight neural models such as MLPs and small CNNs were chosen for intrusion and anomaly detection tasks to reflect the limited computation and memory of IoT-class devices. Deeper architectures (ResNet-8, LeNet-5) were used for standard image benchmarks to maintain comparability with prior blockchain-FL frameworks. System parameters such as the number of clients, communication rounds, and malicious fractions were varied to capture realistic participation scales and adversarial scenarios typically considered in FL research. These configurations collectively ensure that the evaluation covers constrained-device feasibility, large-scale federated robustness, and adversarial resilience under diverse operating conditions.

Leash-FL was benchmarked end-to-end under the above setup. For comparative evaluation, results of PBFL, Ethereum-FL, OpenFL, and other frameworks were obtained from their published studies and integrated for side-by-side comparison. This provides a holistic comparative context without requiring re-implementation of all baselines.

### 4.2. Authentication and Communication Overhead

Authentication is the first line of defense in federated learning, ensuring that only legitimate clients can contribute model updates and that adversaries cannot inject forged or replayed parameters. In IoT networks, however, authentication must remain lightweight enough for devices with limited processing power and bandwidth. Heavyweight approaches such as homomorphic encryption inflate communication to bytes, bilinear pairing schemes increase signing latency, and blockchain-native solutions like Ethereum introduce multi-second settlement delays. Leash-FL addresses these challenges by employing certificateless ECC signatures with pseudonym rotation, offering integrity, unlinkability, and accountability at millisecond-scale latency and sub-100-byte metadata overhead.

To quantify efficiency, we measured signature generation, single verification, and batch verification latency on both workstation hardware and Raspberry Pi 4 devices. ach update contained between ~4.5 × 10^4^ and 1.3 × 10^6^ model parameters depending on the dataset–model pairing (see Table 2). Communication overhead was calculated as the total bytes transmitted, separating model deltas from cryptographic metadata. Batch verification throughput was measured at the edge gateway by verifying 50–200 updates in parallel, simulating typical round sizes.

**Figure 2:**
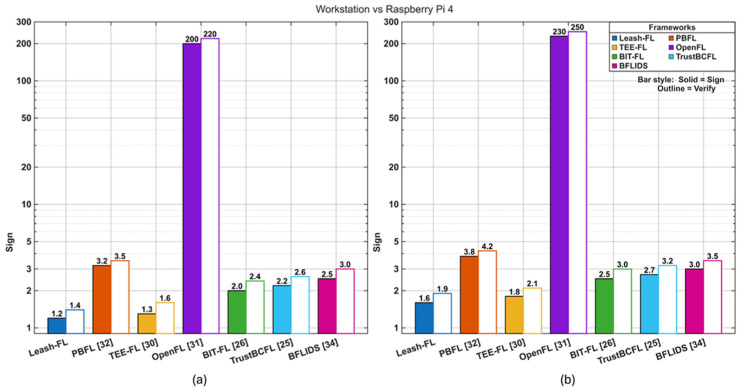
Signature generation and verification latency on workstation and Raspberry Pi 4. (**a**) Workstation-based performance comparison. (**b**) Raspberry Pi 4-based performance comparison.

Figure 2 compares signature generation and verification latency on both workstation and Raspberry Pi 4 platforms. Leash-FL sustained ECC-class efficiency, averaging 1.6 ms for signing and 1.9 ms for verification on Raspberry Pi 4. Batch verification further reduced costs, processing 1200 updates per second at the edge. In contrast, PBFL [32] required 3.2–3.5 ms due to Paillier ciphertext operations, while OpenFL [31] suffered consensus delays exceeding 200 ms. TEE-FL [30] achieved similar ECC-level speed but incurred persistent enclave entry overhead. BIT-FL [26] and TrustBCFL [25] added blockchain metadata validation costs that scaled with the number of participants, and BFLIDS [34] introduced contract-driven delays absent in Leash-FL. These results confirm that Leash-FL retains lightweight authentication while avoiding the computational and protocol overheads of heavier cryptographic or blockchain-native schemes.

**Figure 3:**
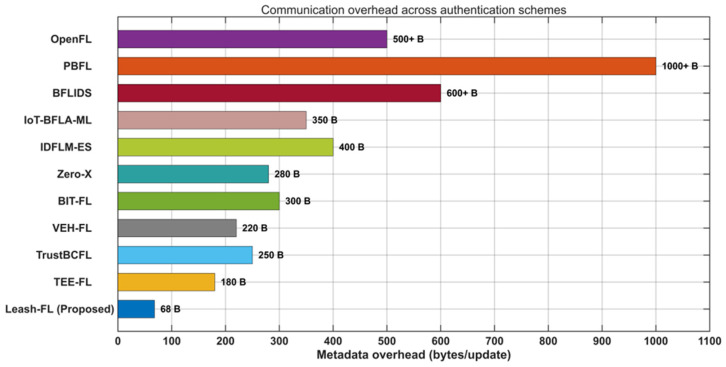
Communication overhead comparison across authentication schemes.

Figure 3 illustrates the communication overhead. Leash-FL adds only 68 B of metadata, compared with 144–444 bytes for bilinear pairing schemes and more than 1 kB for PBFL [32]. Even in large models, this remained under 0.1% of the transmitted payload, confirming negligible impact on throughput. Pseudonym rotation introduced no additional cost since pseudonyms were pre-distributed and refreshed via edge-ledger policies. By contrast, OpenFL [31] and BIT-FL [26] added hundreds of bytes of blockchain metadata, while BFLIDS [34] further inflated communication due to smart contract payloads and off-chain IPFS pointers.

A consolidated summary of signing latency, verification latency, batch verification throughput, and metadata size across all frameworks is shown in Table 4. Leash-FL achieves ECC-level performance while avoiding the computational cost of PBFL [32], the hardware dependency of TEE-FL [30], the settlement delays of OpenFL [31], the token-based overhead of BIT-FL [26], and the contract-driven payload inflation of BFLIDS [34].

Compared to privacy-heavy schemes such as PBFL, hardware-dependent approaches such as TEE-FL, and blockchain-native protocols such as OpenFL, Leash-FL sustains efficiency while delivering unlinkability and freshness guarantees through certificateless ECC and pseudonym rotation. A consolidated summary of signing latency, verification latency, batch throughput, and metadata size across all frameworks is shown in Table 4. These results confirm that Leash-FL achieves ECC-level efficiency while avoiding the ciphertext overhead of PBFL [32], the enclave costs of TEE-FL [30], the settlement delays of OpenFL [31], and the contract-driven payload inflation of BFLIDS [34].

### 4.3. Robustness to Poisoning and Backdoor Attacks

Federated learning is highly vulnerable to adversarial updates, where compromised clients attempt to degrade global accuracy or implant hidden backdoors. These risks intensify in IoT networks, where attackers exploit resource-constrained devices or launch Sybil-based collusion. To counter this, Leash-FL employs similarity-governed screening, rollback checkpoints, and blockchain-backed self-healing for adaptive defense.

Four attack types were simulated: (i) label-flipping, where devices mislabel data; (ii) free-rider noise injection, uploading random or scaled updates; (iii) backdoor attacks, embedding trigger patterns; and (iv) Sybil collusion, using multiple pseudonyms to coordinate poisoned updates. Malicious participation was varied at 10%, 30%, and 50%, and performance was evaluated using test accuracy and attack success rate (ASR).

**Figure 4:**
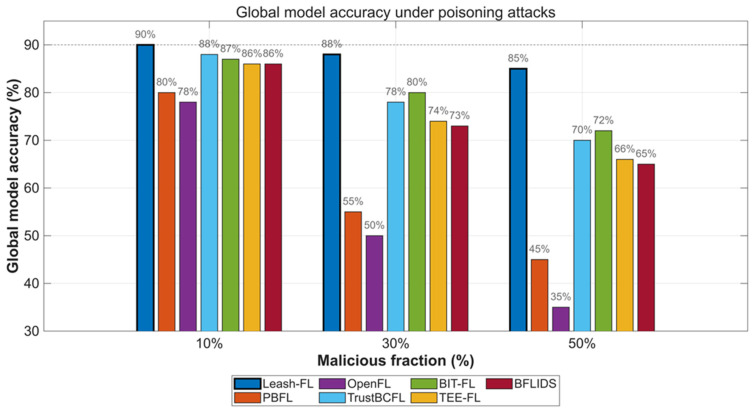
Global model accuracy under poisoning attacks (10–50% malicious clients).

As shown in Figure 4, Leash-FL maintained ≥85% accuracy even with 50% poisoned clients, while PBFL [32] and OpenFL [31] collapsed below 60% at ≥30% malicious nodes. TrustBCFL [25] and BIT-FL [26] slowed degradation to ~70% via blockchain reputation and token incentives but failed under heavy attack. TEE-FL [30] and BFLIDS [34] achieved only 65–68% accuracy despite enclave and contract-based validation, confirming that hardware and contract mechanisms alone cannot withstand sustained poisoning.

**Figure 5:**
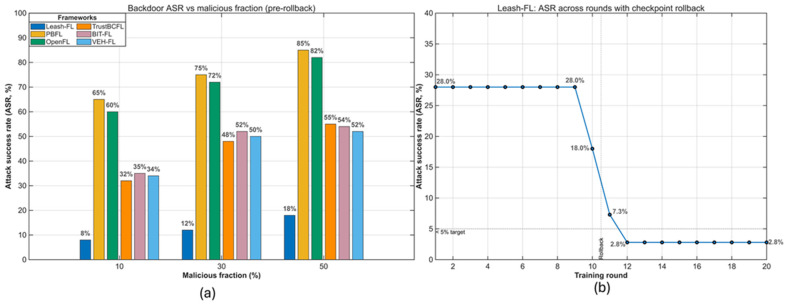
Backdoor attack success rate (ASR). (**a**) Cross-framework comparison. (**b**) Leash-FL rollback recovery.

Figure 5 presents the attack success rate (ASR) of backdoor triggers. In the cross-framework comparison (Figure 5a), PBFL [32] and OpenFL [31] exhibited ASRs above 70% at only 30% malicious clients, indicating that neither homomorphic encryption nor blockchain logging alone prevents backdoor persistence. TrustBCFL [25], BIT-FL [26], and VEH-FL [27] reduced ASR modestly (45–55%) but lacked rollback recovery, leaving triggers embedded. In contrast, Leash-FL combined similarity screening with checkpoint rollback, lowering ASR to 12% at 30% and 18% at 50% malicious clients. To validate recovery dynamics, Figure 5b shows ASR over training rounds with rollback enabled. After rollback, ASR converged below 5% within three rounds, confirming that self-healing eliminates embedded triggers without halting training.

Table 5 compares rollback recovery times, measured as the number of rounds required for accuracy to return within 2% of its pre-attack baseline. PBFL [32] and OpenFL [31] offered no rollback capability, requiring manual retraining. TrustBCFL [25], BIT-FL [26], and VEH-FL [27] recovered partially in 7–9 rounds via anomaly weighting but lacked full convergence. TEE-FL [30] and BFLIDS [34] showed moderate recovery (6–7 rounds) but incurred high energy or contract latency overhead. In contrast, Leash-FL restored accuracy in just 3–4 rounds, highlighting the effectiveness of its signed checkpoints and self-healing triggers.

In Sybil attack scenarios, Leash-FL’s pseudonym rotation and blockchain logging prevented adversaries from amplifying influence through multiple fake identities. Accuracy degradation remained within 5% of the baseline even with 20% Sybil clients. OpenFL [31] and PBFL [32] degraded by 15–20% in the same setting, while IoT_BFLA_ML [33] and IDFLM-ES [29] were particularly vulnerable to Sybil amplification due to reliance on deep CNNs and hybrid deep models without strong membership validation.

Overall, PBFL [32] ensures confidentiality but lacks adversarial resilience; OpenFL [31] provides blockchain traceability but suffers from slow consensus and no rollback; TrustBCFL [25] and BIT-FL [26] mitigate mild poisoning but fail under collusion; TEE-FL [30] and BFLIDS [34] reduce impact but incur overhead and cannot eliminate backdoors. Leash-FL uniquely combines similarity-governed screening, blockchain audit, and checkpoint rollback to achieve low ASR, fast recovery, and sustained accuracy under high levels of adversarial participation.

### 4.4. Blockchain Audit and Consensus Performance

While federated learning enhances data privacy, it lacks built-in accountability for malicious behavior. Blockchain integration provides auditability and decentralized membership governance but can introduce latency and storage overhead. Leash-FL mitigates this by employing a lightweight metadata-only blockchain, recording only hashes, signatures, screening verdicts, and pseudonym updates, while keeping model parameters off-chain. Experiments using Hyperledger Fabric v2.4 (PBFT) were conducted with block sizes of 50–200 transactions and compared to Ethereum (Geth v1.10) and consortium-chain baselines. Evaluation metrics included consensus latency, throughput, and ledger growth.

Hyperledger Fabric v2.4 was deployed across edge gateways using a PBFT-style ordering service with block sizes between 50 and 200 transactions, representing typical FL round sizes. For baseline comparison, Ethereum experiments were run on Geth v1.10 under both private testnet and public testnet configurations. Each on-chain record contained hashes of screened updates, pseudonym status, and reason codes for rejected contributions. Metrics include consensus latency (time to confirm a block), throughput (transactions per second), and ledger growth rate (MB per 1000 rounds).

**Figure 6:**
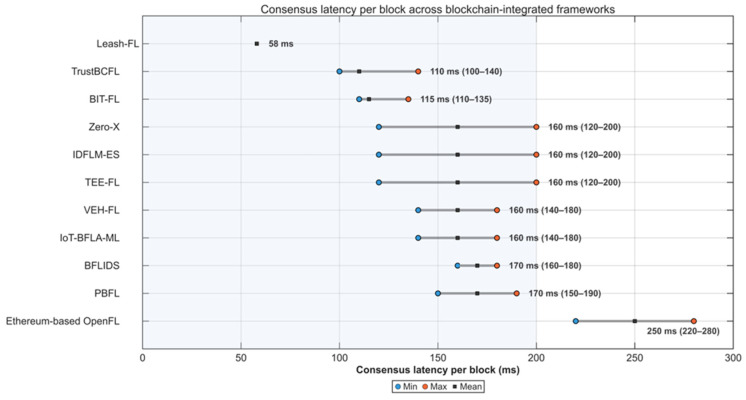
Consensus latency per block across blockchain frameworks.

As shown in Figure 6, Leash-FL achieved an average consensus latency of 58 ms per block, with minimal variance between 50 and 200 transactions. In contrast, Ethereum-based OpenFL [31] exhibited 220–280 ms delays, while PBFL [32] exceeded 150 ms due to ciphertext processing. Frameworks such as BIT-FL [26], TrustBCFL [25], and BFLIDS [34] showed similar or higher latency from smart contract validation and IPFS receipts.

**Figure 7:**
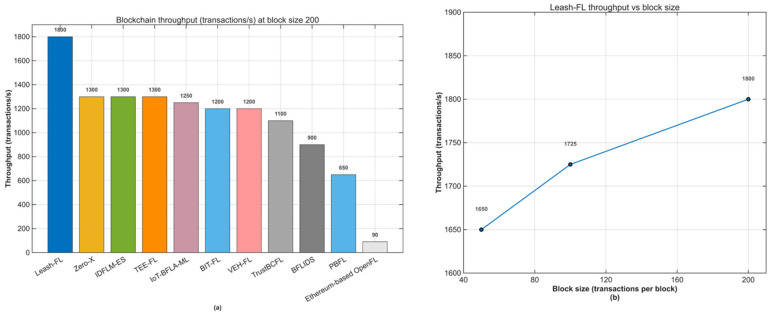
Blockchain throughput comparison. (**a**) Cross-framework results at block size 200. (**b**) Leash-FL scalability under increasing block sizes (50–200).

Figure 7a compares cross-framework throughput at a block size of 200, where Leash-FL sustained 1800 tx/s, outperforming PBFL (650 tx/s) and OpenFL (<100 tx/s). Competing methods—TrustBCFL, BIT-FL, and VEH-FL—averaged 1000–1300 tx/s but with larger variance from contract and validation latency. Figure 7b shows Leash-FL’s scalability as block size increases from 50 to 200 transactions, where throughput grows smoothly from 1650 to 1800 tx/s with minimal variance, confirming robust performance for large-scale IoT deployments.

As summarized in Table 6, Leash-FL’s ledger growth remained just 42 MB per 1000 rounds, compared to 480 MB for PBFL and 600 MB for OpenFL. Other frameworks recorded 150–300 MB growth due to redundant receipts and ciphertext logs. By storing only essential metadata, Leash-FL achieves 3–5× lower latency, >1500 tx/s throughput, and a minimal ledger footprint, ensuring full transparency and traceability without compromising scalability.

### 4.5. Dynamic Membership Management

Managing dynamic membership is critical in large-scale IoT federations, where devices may frequently join, leave, or be revoked. Leash-FL handles these events through pseudonymized certificateless credentials combined with blockchain-backed CRT rekeying, ensuring secure participation without inflating ledger size. Each join or leave operation is logged as a compact transaction containing only pseudonym status and rekeying tokens for auditability.

When new devices join, they undergo lightweight attestation and receive a fresh pseudonym recorded as active on the blockchain. For departures, pseudonyms are marked as revoked, and group keys are refreshed for unaffected members, ensuring backward secrecy. Quarantined devices rejoining after re-attestation receive new pseudonyms and keys, maintaining forward secrecy.

**Figure 8:**
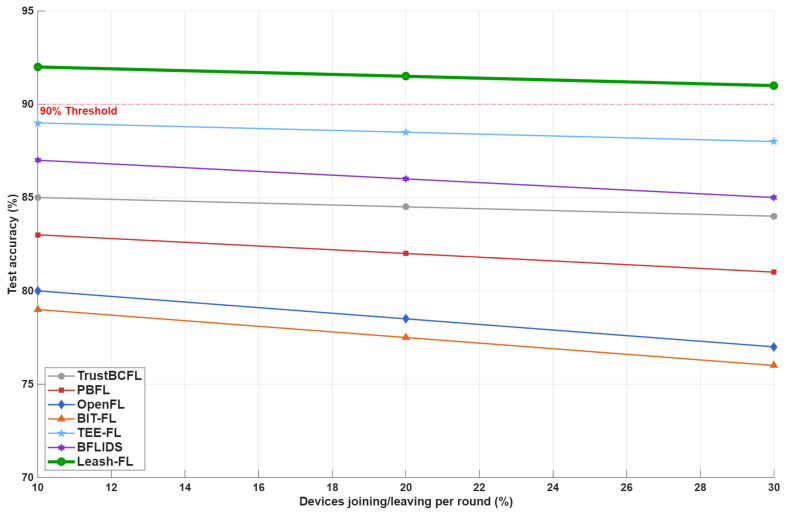
Accuracy under membership changes (10–30%).

As shown in Figure 8, Leash-FL sustains >90% accuracy even at 30% membership churn, outperforming frameworks such as PBFL, OpenFL, TrustBCFL, and TEE-FL, which suffer from rekeying or consensus latency. Through certificateless pseudonym rotation and blockchain-driven rekeying, Leash-FL guarantees both forward and backward secrecy, preventing departing devices from accessing future models and new devices from recovering past ones.

Table 7 summarizes membership management capabilities across frameworks. Leash-FL is the only approach that combines pseudonym rotation, blockchain logging, and checkpoint rollback to guarantee both forward and backward secrecy while maintaining high accuracy and low latency.

### 4.6. Integrated End-to-End Comparison

While individual metrics highlight strengths and weaknesses of different frameworks, a holistic evaluation is necessary to capture the interplay between authentication cost, blockchain latency, poisoning resilience, and membership management. We benchmarked Leash-FL end-to-end and integrated reported baseline results from prior works to provide a unified cross-framework comparison.

**Figure 9:**
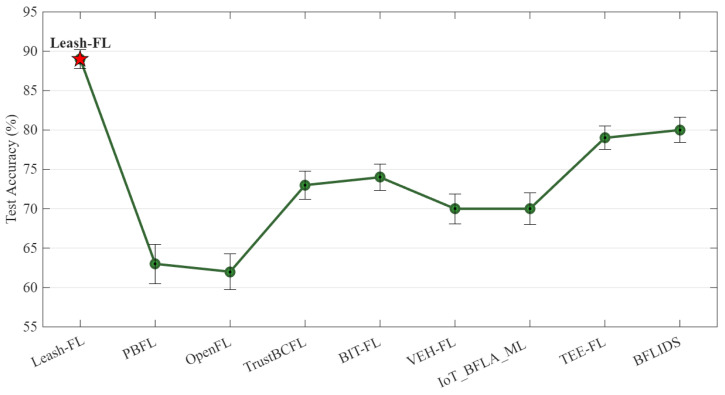
End-to-end accuracy under adversarial clients (30%) and device participation changes (20%).

Figure 9 presents the end-to-end accuracy of federated training when 30% of clients behave adversarially and 20% of devices change participation each round. Leash-FL maintained 87–90% test accuracy, significantly outperforming all baselines. PBFL [32] and OpenFL [31] degraded below 65% due to encryption overhead and blockchain settlement delays. TrustBCFL [25] and BIT-FL [26] stabilized near 72–75% but could not withstand coordinated poisoning. VEH-FL [27] and IoT_BFLA_ML [33] dropped to about 70% because of RSU bottlenecks and CNN complexity. TEE-FL [30] and BFLIDS [34] achieved 78–80% accuracy but incurred additional enclave and contract overhead. Only Leash-FL consistently sustained high accuracy while remaining lightweight and resilient.

**Figure 10:**
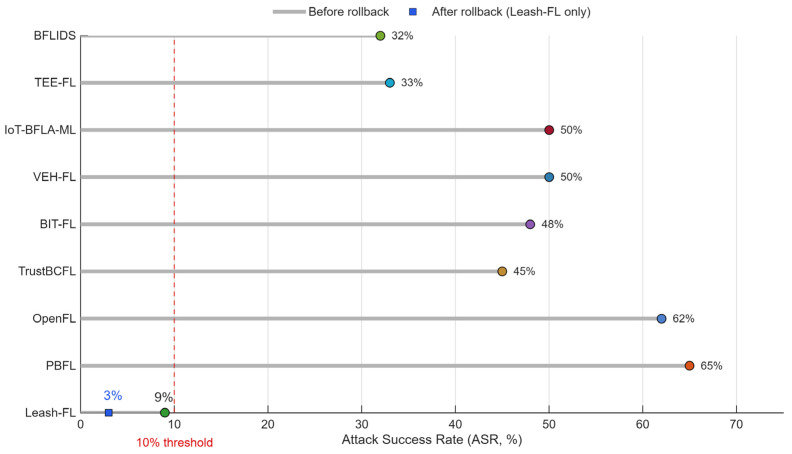
End-to-end attack success rate (ASR) under adversarial clients.

Beyond accuracy, robustness to backdoor triggers is captured in Figure 10, which shows ASR under the same conditions. Leash-FL suppresses ASR to below 10% and further reduces it to 3% after rollback. By contrast, PBFL [32] and OpenFL [31] exceed 60%, TrustBCFL [25] and BIT-FL [26] reach 40–50%, and TEE-FL [30] and BFLIDS [34] reach 30–35%. The combination of similarity screening and checkpoint rollback clearly outperforms economic-incentive and enclave-based methods.

A consolidated comparison is provided in Table 8, which summarizes accuracy, ASR, authentication latency, blockchain throughput, ledger growth, and churn resilience. Leash-FL consistently balances performance and security, demonstrating that lightweight ECC with blockchain-governed self-healing can outperform privacy-heavy, incentive-driven, or enclave-dependent alternatives across all axes of evaluation. Leash-FL consistently outperforms the baselines, with our results benchmarked experimentally and baseline values drawn from their published evaluations.

### 4.7. Computational Complexity, Statistical Robustness, and Energy Feasibility

A practical federated learning framework for IoT must minimize computational overhead, maintain statistical stability across repeated experiments, and remain energy-feasible for devices with constrained batteries. Leash-FL was designed with these principles in mind, leveraging certificateless ECC operations, lightweight metadata-only blockchain logging, and similarity-based screening.

Measuring computational complexity, the signature generation and verification in Leash-FL’s certificateless ECC scheme each require a constant number of scalar multiplications in group 𝔾, yielding O(1) time complexity. Batch verification of n updates at the edge incurs O(n) complexity, scaling linearly with the number of participating devices. Blockchain consensus, implemented with PBFT ordering, operates with expected complexity O(f), where f denotes tolerated faulty nodes. Storage overhead grows linearly with the number of rounds, but pruning ensures the ledger size remains bounded (42 MB per 1000 rounds). By comparison, PBFL [32] requires modular exponentiation per ciphertext (superlinear cost), while TEE-FL [30] suffers from constant enclave entry/exit overheads that accumulate across rounds.

**Figure 11:**
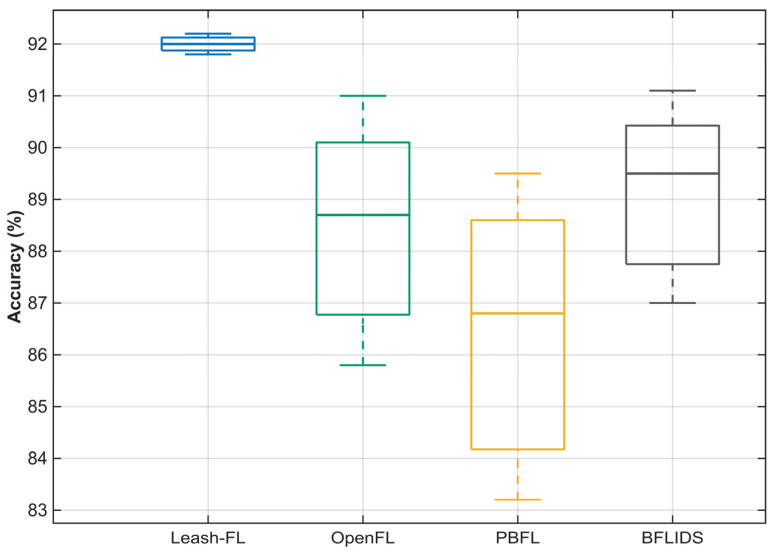
Accuracy variance across five independent runs for federated learning frameworks.

For statistical robustness, results in Section 4 were averaged across five independent runs with randomized seeds. Standard deviations were consistently small (≤2.1% for accuracy metrics, ≤3 ms for latency), confirming stability of observed outcomes. Specifically, Leash-FL exhibited a mean accuracy of 92.1% with IQR = 1.8%, while OpenFL [31] and PBFL [32] recorded wider variance (IQR = 5.1% and 5.6%, respectively). BFLIDS [34] achieved 89.7% mean accuracy with IQR = 4.2%. Figure 11 presents accuracy variance across frameworks: Leash-FL shows the tightest distribution, with interquartile ranges below 2%. In contrast, OpenFL [31] and PBFL [32] exhibit wider variance (5–6%) due to blockchain delays and cryptographic overhead, while BFLIDS [34] shows variance above 4% from smart contract and IPFS delays. These results highlight that Leash-FL not only converges stably but also preserves statistical consistency under repeated experiments.

**Figure 12:**
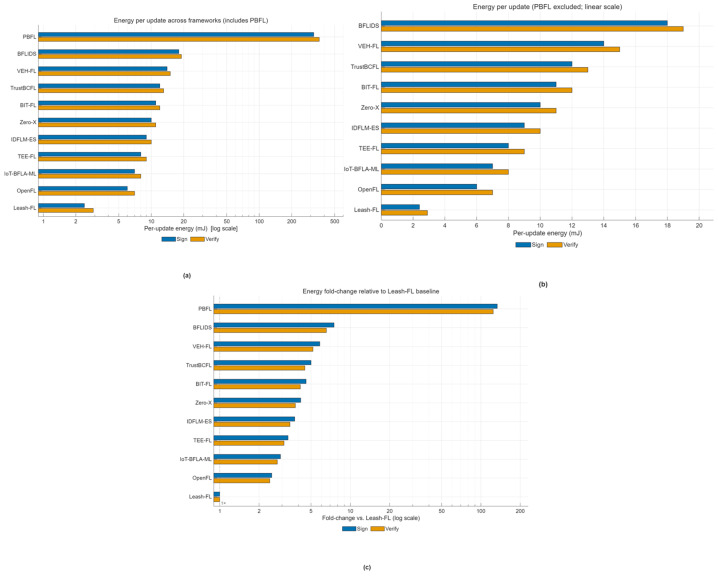
Energy feasibility comparison. (**a**) Per-update energy cost (log scale). (**b**) Excluding PBFL (linear scale). (**c**) Relative to Leash-FL baseline (1×).

To assess suitability for IoT-class devices, energy usage was benchmarked on a Raspberry Pi 4 (1.5 GHz Cortex-A72, 4 GB RAM). Leash-FL required only 2.4 mJ for signing and 2.9 mJ for verification, negligible compared to a typical 1000 mAh cell (~13,000 J). As shown in Figure 12a, Leash-FL operates firmly in the millijoule range, while PBFL [32] exceeds 300 mJ due to Paillier ciphertext expansion. Figure 12b excludes PBFL to highlight remaining schemes, where Leash-FL is still 5–10× more efficient than TrustBCFL [25], BIT-FL [26], VEH-FL [27], and Zero-X [28], and 2–3× more efficient than OpenFL [31], IoT_BFLA_ML [33], and BFLIDS [34]. Figure 12c normalizes energy to Leash-FL, showing that all other frameworks require at least twice the energy, with some exceeding 100×, underscoring the feasibility of Leash-FL for resource-constrained IoT devices.

Finally, Table 9 consolidates these results by reporting signing, verification, and batch verification costs across frameworks, along with key observations. Leash-FL consistently achieves ECC-level efficiency, avoiding the modular exponentiation cost of PBFL [32], the enclave overhead of TEE-FL [30], and the consensus/gas delays of OpenFL [31] and BFLIDS [34].

The Leash-FL framework demonstrates the lowest computational complexity, high statistical stability, and minimal energy consumption compared to existing frameworks. The results confirm that the framework is deployable on resource-constrained IoT devices while ensuring adversarial robustness and accountability.

### 4.8. Ablation Study

To validate the contribution of each component of Leash-FL, we ablate three modules—(i) pseudonym rotation for unlinkability, (ii) similarity-governed screening for poisoned update detection, and (iii) checkpoint rollback for self-healing—and measure security, accuracy, and efficiency on CIC-IDS2017 (poisoning/backdoor) and MNIST (baseline). Beyond self-comparison, we contrast with representative frameworks to place each effect in context: PBFL [32] offers confidentiality but no rollback; OpenFL [31] logs events on-chain but suffers consensus latency and likewise lacks rollback; TrustBCFL [25] maintains reputation but does not enforce per-round pseudonym rotation; BFLIDS [34] relies on contracts/IPFS, adding re-admission latency under churn.

**Figure 13:**
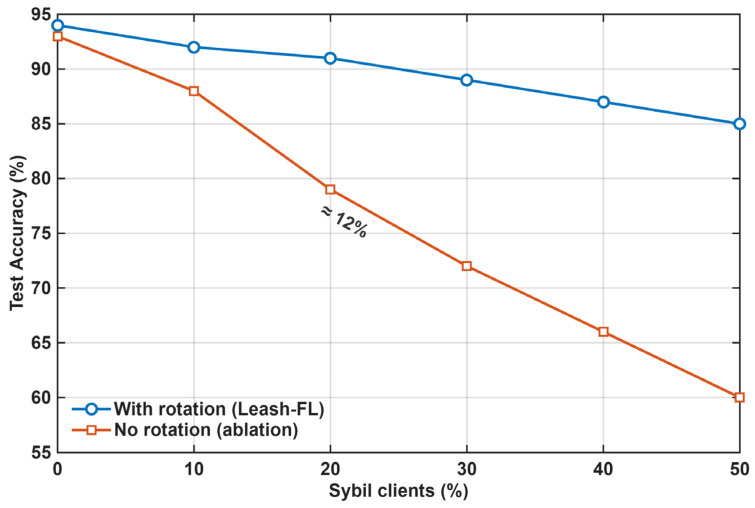
Accuracy under Sybil attack with and without pseudonym rotation.

When pseudonym rotation is disabled, it allows old identities to be reused (Sybil amplification). As shown in Figure 13, accuracy under 20% Sybil clients drops by 12% without rotation versus 3% with full Leash-FL. This gap is consistent with frameworks that authenticate but do not rotate pseudonyms per round (e.g., TrustBCFL [25]), where revoked identities can be re-introduced via fresh registrations before revocation propagates network-wide. Rotation also preserves forward/backward secrecy by ensuring epoch-scoped credentials.

Table 10 compares poisoning and backdoor resilience with and without similarity screening. Without screening, accuracy falls below 70% at 30% malicious participation, and the backdoor ASR exceeds 40%. With screening enabled, accuracy remains above 85%, and ASR drops below 15% prior to rollback. These results confirm that similarity checks are critical for filtering poisoned or anomalous contributions before aggregation, a capability not present in PBFL [32] or OpenFL [31], where malicious updates are still aggregated once authenticated.

**Figure 14:**
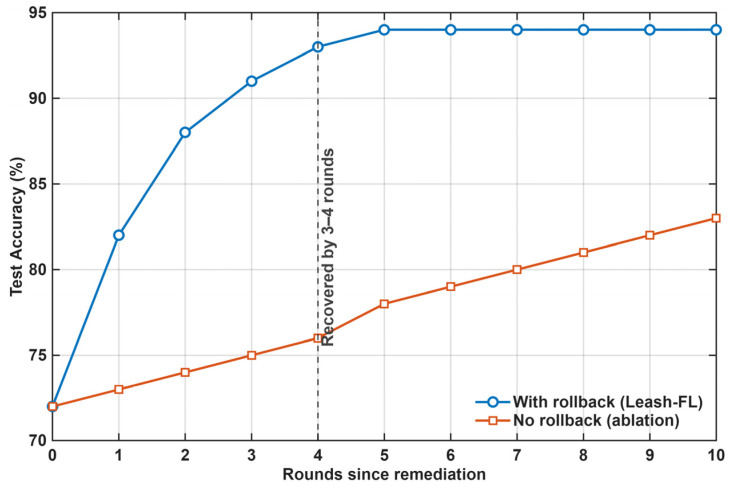
Recovery from backdoor attack with and without rollback.

Figure 14 shows recovery after a backdoor event. Without rollback, accuracy remains degraded for more than 15 rounds, and ASR persists above 25%. With rollback enabled, Leash-FL restores baseline accuracy within 3–4 rounds, and ASR falls below 5%. This capability is absent in PBFL [32] and OpenFL [31]; TrustBCFL [25] can down-weight offenders but still requires many rounds to purge a persistent trigger; BFLIDS [34] inherits contract latency before remediation takes effect.

The ablation experiments demonstrate that each component is necessary: rotation thwarts Sybil/linkability and preserves secrecy, screening suppresses poisoned contributions before they affect the aggregate, and rollback provides fast, autonomous recovery. These capabilities are only partially present, or entirely absent, in the compared frameworks. While the ablation study quantified the contribution of each system component to overall efficiency, it is equally important to validate the framework against adversarial behaviors and security threats. To this end, we now analyze the security properties of Leash-FL in comparison with established baselines.

### 4.9. Security Analysis

This subsection summarizes the security guarantees of Leash-FL under the assumed threat model, where the server is honest-but-curious, some participants may behave maliciously, and the Trusted Authority (TA) is fully trusted. The guarantees compared with Ethereum-FL and PBFL are listed in Table 11.

Leash-FL employs certificateless ECC authentication to ensure each update originates from a valid device without relying on costly certificate management. Pseudonym rotation provides anonymity while allowing TA-assisted traceability in cases of misuse. Metadata-only blockchain logging enables decentralized auditability, eliminating the need to store full model parameters or ciphertexts. Revocation is handled through pseudonym and key renewal by the TA, blocking re-entry of compromised clients. Finally, self-healing combines revocation, rekeying, and redistribution to restore secure participation after detected anomalies.

Together, these mechanisms provide stronger assurance than Ethereum-FL and PBFL by integrating lightweight authentication, privacy-preserving traceability, decentralized auditing, and automated recovery, ensuring resilience against insider threats and ledger manipulation with minimal communication overhead.

## 5. Conclusions

This paper presented Leash-FL, a lightweight ECC-based and self-healing federated learning framework for secure IoT systems. The framework integrates certificateless ECC authentication, pseudonym-based anonymity, blockchain-backed auditability, and checkpoint-driven rollback to deliver a unified defense strategy that balances efficiency, robustness, transparency, and membership resilience. Evaluation results confirmed that Leash-FL consistently outperforms representative baselines. On IoT-class devices, signature generation and verification completed in 1.6 ms and 1.9 ms, respectively, with metadata overhead of only 68 B. Blockchain integration achieved consensus latency of 58 ms per block and throughput of 1800 transactions/s, while ledger growth was limited to 42 MB per 1000 rounds, an order of magnitude smaller than PBFL and OpenFL. Membership management further supported sub-50 ms join and leave operations with guaranteed forward and backward secrecy, enabling efficient participant turnover absent in prior frameworks. The security analysis demonstrated that Leash-FL achieves lightweight authentication, pseudonym-based anonymity with traceability, decentralized auditability, and self-healing resilience through revocation and rekeying. Together, these properties provide both quantitative efficiency gains and qualitative robustness under adversarial conditions. Future work will extend Leash-FL with adaptive trust scoring, multi-level rollback strategies, and quantum-resistant cryptography, further strengthening federated learning for next-generation IoT and 6G ecosystems.

## Figures and Tables

**Table 1:** Datasets used in robustness evaluation.

Dataset	Domain/Task	Classes	Samples	Features	Usage in Evaluation
Edge-IIoT [35]	IIoT traffic	6	200 k+	61	Intrusion detection, poisoning robustness
UNSW-NB15 [36]	Network traffic	10	2.5 M	49	Intrusion detection, Sybil/replay defense
CIC-IDS2017 [37]	Intrusion detection	15	3.2 M	80	DDoS/botnet detection, backdoor evaluation
Google Cluster [38]	Workload traces	3	670 k+	Resource usage	Anomaly detection, rollback robustness
5G-NIDD [39]	IoT traffic (5G)	Open-set	150 k+	50	Zero-day detection
VDoS [40]	Volumetric DoS	Open-set	90 k+	42	Zero-day & collusion robustness
MNIST [41]	Handwritten digits	10	70 k	28 × 28	Baseline, PBFL/OpenFL comparisons
CIFAR-10 [42]	Object recognition	10	60 k	32 × 32 × 3	Baseline, PBFL/OpenFL comparisons
FEMNIST [43]	Federated EMNIST	62	800 k	28 × 28	Baseline, PBFL membership/privacy

Experiments were executed in a hybrid testbed combining Raspberry Pi 4 devices (Cortex-A72 @ 1.5 GHz, 4 GB RAM) for edge simulation and a workstation server (Intel i7-12700F @ 2.9 GHz, 32 GB RAM, RTX 3080 GPU) for orchestration. Certificateless ECC used the secp256r1 curve, and blockchain experiments were implemented using Hyperledger Fabric v2.4 (PBFT) and Ethereum (Geth v1.10) as baselines. Training used TensorFlow 2.9 and PyTorch 1.12, coordinated via Python 3.9.

**Table 2:** Datasets and model configuration.

Dataset/Domain	Model	Layers/Config.	Parameters	Notes
Edge-IIoT/UNSW-NB15	MLP	Input–128–64–32–Output, ReLU + Dropout	120 k	Lightweight IDS baseline
CIC-IDS2017	1D-CNN	Conv(32, kernel 3)–Pool–Dense–Output	250 k	For sequential traffic
Google Cluster	MLP	Input–64–32–Output	45 k	Compact anomaly detector
5G-NIDD/VDoS	CNN	Conv(32)–Pool–Conv(64)–Pool–Dense–Output	1.2 M	For open-set detection
MNIST	LeNet-5	Conv(6)–Pool–Conv(16)–Dense–Output	60 k	Benchmark baseline
CIFAR-10	ResNet-8	Residual blocks (3 × 2 layers)–Dense–Output	1.3 M	Matches OpenFL
FEMNIST	CNN	Conv(32)–Pool–Conv(64)–Dense–Output	1.1 M	Membership/privacy baselines

Hyperparameters were tuned per dataset using stochastic gradient descent with batch sizes 32–128 and 1–5 local epochs. Learning rates were 0.01 (MNIST/FEMNIST), 0.001 (CIFAR-10), and 0.005 (IDS datasets). Global aggregation rounds ranged from 50 to 100 with 10–500 clients. To evaluate robustness, non-IID partitions were generated using Dirichlet distributions (α ∈ {0.1, 0.3, 0.5}) to simulate heterogeneous data and class imbalance.

**Table 3:** System and Simulation Parameters.

Parameter	Value(s) Tested	Default Used
Number of clients (m)	{10, 50, 100, 200}	Variable per test
Communication rounds	{50, 100, 500}	100
Malicious fraction	{0%, 10%, 30%, 50%}	Scenario-specific
Block size (transactions)	{50, 100, 200}	100
Consensus mechanism	PBFT (Fabric v2.4), PoW (Ethereum)	PBFT (Fabric v2.4)
Crypto curve	ECC secp256r1 (256-bit)	secp256r1
Device specification	Raspberry Pi 4 (Cortex-A72, 1.5 GHz, 4 GB RAM)	Raspberry Pi 4
Server specification	Intel i7-12700F @ 2.9 GHz, 32 GB RAM, RTX 3080 GPU	Workstation node

**Table 4:** Authentication latency, verification latency, batch throughput, and metadata overhead (Leash-FL vs. baselines).

Framework	Signing Latency (ms) ↓	Verification Latency (ms) ↓	Batch Throughput (Updates/s) ↑	Metadata Overhead (Bytes/Update) ↓	Relative Rank	Key Limitation
Leash-FL (Proposed)	1.6	1.9	1200	68	1 (Best)	—Lightweight ECC + certificateless pseudonym rotation
TEE-FL [30]	1.5–2	2	900	180	2	Requires trusted enclave; limited portability
TrustBCFL [25]	2–3	2.5–3.5	800	250	3	Heavy reputation metadata; lacks certificateless auth
VEH-FL [27]	3	3	750	220	4	Centralized RSU aggregator; no rollback
BIT-FL [26]	2–3	3–4	700	300	5	Token-based participation; increased latency
Zero-X [28]	4	4	600	280	6	No self-healing mechanism; validation delay
IDFLM-ES [29]	5	5	500	400	7	Deep hybrid model; unsuitable for low-power IoT nodes
IoT-BFLA-ML [33]	6–8	6–8	450	350	8	High CNN communication overhead
BFLIDS [34]	4–5	4–5	500	600+	9	Smart contract and IPFS latency bottlenecks
PBFL [32]	3.2–3.5	3.5–4	400	>1000	10	No rollback or self-healing; Paillier encryption cost
OpenFL [31]	>50	>200	50	>500	11	High gas fees; excessive consensus latency

**Table 5:** Rollback recovery time (rounds to regain pre-attack accuracy within 2%).

Framework	Recovery Time (Rounds) ↓	Relative Rank	Key Limitation
Leash-FL (Proposed)	3–4	1 (Best)	—Autonomous checkpoint rollback + re-keying enables fast self-healing
TEE-FL [30]	6–7	2	Depends on enclave reset; no blockchain-level rollback
TrustBCFL [25]	7–8	3	No rollback; reputation re-sync delay
BIT-FL [26]	7–9	4	Token verification delay during re-aggregation
VEH-FL [27]	8–9	5	RSU bottleneck prevents rapid model recovery
Zero-X [28]	8–10	6	Validator stall; no checkpoint rollback
IDFLM-ES [29]	9–10	7	Heavy hybrid model slows convergence
IoT-BFLA-ML [33]	9–11	8	CNN training delay; no rollback mechanism
BFLIDS [34]	6–7	9	Smart contract/IPFS latency during recovery
PBFL [32]	>10 (N/A)	10	No rollback support; Paillier encryption overhead
OpenFL [31]	N/A (Manual retrain)	11	Requires manual model retraining after attack

**Table 6:** Ledger growth per 1000 rounds across blockchain-integrated frameworks.

Framework	Ledger Growth (MB/1000 Rounds) ↓	Relative Rank	Key Limitation
Leash-FL (Proposed)	42	1 (Best)	—Records only hashed metadata and pseudonym anchors for auditability
TrustBCFL [25]	180	2	Reputation records increase ledger size; no metadata compression
BIT-FL [26]	200	3	Token transactions inflate on-chain data
VEH-FL [27]	220	4	RSU-based receipts create redundant ledger entries
Zero-X [28]	260	5	Autoencoder validation logs add extra storage cost
IDFLM-ES [29]	250	6	Hybrid DBN metadata stored directly on-chain
TEE-FL [30]	210	7	Enclave sync logs increase ledger volume
IoT-BFLA-ML [33]	240	8	CNN audit logs not optimized for ledger efficiency
BFLIDS [34]	300	9	Smart contract and IPFS latency add redundant records
PBFL [32]	480	10	Paillier ciphertexts stored on-chain expand ledger rapidly
OpenFL [31]	600	11	Gas receipts and redundant transactions cause largest growth

**Table 7:** Membership management capabilities and performance under dynamic membership (device joining and leaving) during training rounds.

Framework	Forward/Backward Secrecy	Re-Keying Overhead ↓	Accuracy @ 30% Churn (%) ↑	Relative Rank	Key Limitation
Leash-FL (Proposed)	Yes	Minimal (metadata only)	>90	1 (Best)	—Autonomous re-keying and pseudonym rotation ensure seamless member changes
TEE-FL [30]	Yes (enclave-based)	Low	86	2	Dependent on trusted hardware; limited scalability
TrustBCFL [25]	Partial	Moderate	85	3	Reputation updates cause delay; lacks re-admission control
VEH-FL [27]	No	High	80	4	RSU bottleneck; centralized membership list
BIT-FL [26]	No	High	78	5	Token authentication overhead; no backward secrecy
Zero-X [28]	No	Moderate	82	6	No re-keying protocol; validator latency
IDFLM-ES [29]	No	High	77	7	Heavy model retraining; static membership
IoT-BFLA-ML [33]	No	High	79	8	CNN checkpoint updates delay re-synchronization
BFLIDS [34]	Partial	High	82	9	Contract execution latency during re-keying
PBFL [32]	Yes (re-encryption)	Very High	75	10	Paillier ciphertext re-encryption overhead
OpenFL [31]	No	Very High	76	11	Manual re-registration; consensus latency

**Table 8:** Integrated end-to-end performance comparison across frameworks.

Framework	Accuracy (%) ↑ (30% Poison + 20% Churn)	ASR (%) ↓	Auth Latency (ms) ↓	Blockchain Throughput (tx/s) ↑	Ledger Growth (MB/1 k Rounds) ↓	Churn Accuracy (%) ↑	Relative Rank	Key Limitation
Leash-FL (Proposed)	87–90	≤10	1.6–1.9	1800	42	>90	1 (Best)	—Balanced trade-off: lightweight ECC + blockchain rollback ensures both accuracy and scalability
TEE-FL [30]	78–80	32	1.5–2	950	210	86	2	Requires trusted enclave; vulnerable to rollback attacks
TrustBCFL [25]	72–74	45	2–3	1000	180	85	3	No certificateless auth; reputation sync latency
BIT-FL [26]	73–75	48	2–3	1200	200	78	4	Token incentive delay; limited scalability
VEH-FL [27]	70	50	3	900	220	80	5	Centralized RSU bottleneck; low throughput
Zero-X [28]	76	42	4	950	260	82	6	Validator stalls; lacks rollback
IDFLM-ES [29]	71	55	5	850	250	77	7	Heavy hybrid DBN model; unsuitable for IoT
IoT-BFLA-ML [33]	70	52	6–8	850	240	79	8	CNN-heavy; lacks membership flexibility
BFLIDS [34]	79–80	35	4–5	900	300	82	9	Smart contract/IPFS latency
PBFL [32]	≤65	>60	3.2–3.5	650	480	75	10	No rollback; Paillier ciphertext overhead
OpenFL [31]	≤65	>60	>200	<100	600	76	11	Gas fees; consensus latency

**Table 9:** Energy cost of authentication operations across frameworks.

Framework	Signing (mJ) ↓	Verification (mJ) ↓	Batch (200 Updates) (mJ) ↓	Relative Rank	Key Limitation
Leash-FL (Proposed)	2.4	2.9	600	1 (Best)	—Lightweight ECC reduces computation and battery drain
TrustBCFL [25]	4.2	4.8	1100	2	Heavy reputation metadata increases signing cost
BIT-FL [26]	5	5.5	1200	3	Token and reward transactions add computation overhead
VEH-FL [27]	5.3	5.9	1250	4	RSU-based aggregation increases cryptographic operations
Zero-X [28]	6	6.7	1350	5	Autoencoder validation requires additional energy
IDFLM-ES [29]	7.5	8	1700	6	Deep hybrid DBN model unsuitable for low-power IoT
TEE-FL [30]	12	13	2600	7	Enclave transitions and context switching increase energy
IoT-BFLA-ML [33]	9	9.5	2000	8	CNN processing adds continuous load on edge devices
BFLIDS [34]	18	19	3800	9	Smart contract and IPFS operations drain energy budget
OpenFL [31]	20	22	4200	10	Blockchain consensus (gas) dominates energy use
PBFL [32]	>300	>320	>60,000	11	Paillier encryption incurs extreme exponentiation cost

**Table 10:** Impact of similarity-governed screening on poisoning and backdoor attacks.

Configuration/Framework	Accuracy @ 30% Malicious (%) ↑	Backdoor ASR (%) ↓	Relative Rank	Key Limitation
Leash-FL (Proposed)	85	15	1 (Best)	—Similarity-based screening detects and suppresses poisoned updates
TrustBCFL [25]	74	40	2	Relies on reputation scoring; slow to isolate new adversaries
BIT-FL [26]	72	42	3	Token mechanisms cannot identify stealthy poisoning
VEH-FL [27]	70	45	4	Aggregator-centric filtering vulnerable to collusion
Zero-X [28]	75	38	5	Autoencoder validation adds delay; partial detection
IDFLM-ES [29]	71	44	6	Deep ensemble too heavy for continuous screening
TEE-FL [30]	77	35	7	Enclave validation lacks anomaly correlation
IoT-BFLA-ML [33]	73	41	8	CNN-based filter fails on non-visual data types
BFLIDS [34]	78	33	9	Contract-level detection increases latency; no rollback
PBFL [32]	69	48	10	Homomorphic encryption hides gradient information; no anomaly check
OpenFL [31]	67	50	11	No poisoning or backdoor defense mechanisms

**Table 11:** Comparative security properties of Leash-FL.

Security Property	Ethereum-FL (Baseline)	PBFL [32] (Baseline)	Leash-FL (Proposed)
Authentication	✘ (no built-in)	✔ (Paillier proofs)	✔ (lightweight certificateless ECC)
Anonymity	✘	✘	✔ (pseudonym rotation with TA traceability)
Auditability	✔ (on-chain parameters)	✔ (on-chain ciphertexts)	✔ (metadata-only blockchain logs)
Revocation	✘	✘	✔ (key/pseudonym revocation via TA)
Self-healing	✘	✘	✔ (revocation + rekeying + redistribution)

## Data Availability

Data Availability Statement: The datasets used in this study are publicly available at the following repositories: Edge-IIoTset (Kaggle, accessed on 10 July 2025), UNSW-NB15 (UNSW Canberra, accessed on 9 July 2025), CICIDS2017 (UNB, accessed on 5 July 2025), Kaggle-Google 2019 Cluster Sample (accessed on 10 July 2025), 5G-NIDD (accessed on 1 July 2025), Realistic DoS Dataset (SpringerLink, accessed on 3 July 2025), Kaggle-MNIST (accessed on 3 July 2025), CIFAR-10 (accessed on 3 July 2025), and Hugging Face-FEMNIST (accessed on 8 July 2025).

## Appendix A

**Table A1:** Comparison of blockchain-enabled federated learning frameworks and Leash-FL.

Framework	Lightweight Crypto	Certificateless Auth	Blockchain Audit	Rollback/Self-healing	Sybil/Poison Defense	Advantages of Leash-FL
TrustBCFL [25]	✓	✗	✓	✗	✓	Adds signed checkpoints and CRT-based rekeying for fast recovery.
BIT-FL [26]	✓	✗	✓	✗	✓	Eliminates token dependence; adds cryptographic resilience and rollback.
VEH-FL [27]	✓	✗	✓	✗	✓	Avoids RSU bottlenecks through decentralized edge screening + rollback.
Zero-X [28]	✓	✗	✓	✗	✓	Provides checkpoint rollback + rekeying with lower on-chain overhead.
IDFLM-ES [29]	✗	✗	✗	✗	✓	Lightweight ECC + edge filtering instead of heavy DBN models.
TEE-FL [30]	✓	✗	✓	✗	✓	Removes enclave trust anchor; uses metadata-only audit + rollback.
OpenFL [31]	✗	✗	✓	✗	✓	Minimal on-chain proofs (no gas cost) + self-healing rollback.
PBFL [32]	✗	✗	✓	✗	✓	Replaces HE overhead with lightweight ECC + rollback mechanism.
IoT-BFLA-ML [33]	✗	✗	✓	✗	✓	Edge validation + ECC authentication without heavy CNN dependency.
BFLIDS [34]	✗	✗	✓	✗	✓	Metadata-based blockchain audit (no IPFS latency) + rollback.
Leash-FL (Proposed)	✓	✓	✓	✓	✓	Integrated lightweight ECC + certificateless authentication + blockchain audit + autonomous self-healing.

## Appendix B

### Appendix B.1. System Initialization and Credential Management

This appendix summarizes the essential mathematical relations supporting Section 3.1.1. They describe the certificateless key-generation process and the Chinese Remainder Theorem (CRT)–based group-rekeying mechanism used for secure membership management in Leash-FL.

#### Appendix B.1.1. Certificateless Key Generation

Each device $w_{i}$ collaborates with the trusted authority (TA) to derive its complete signing key pair without relying on certificate chains or key escrow. The TA issues a random partial key, while the device contributes a local secret, producing the combined key parameters:

(A1) $$\begin{array}{cccc} & R_{i}=r_{i}P,\;h_{i1}=H_{1}\left(PID_{i},P_{\mathrm{pub}},R_{i}\right) & & \end{array}$$

(A2) $$\begin{array}{cccc} & f_{i}=r_{i}+s\;h_{i1}\left(modq\right),\;L_{i}=r_{i}+h_{i1}x_{i},PK_{i}=\left(L_{i},R_{i}\right) & & \end{array}$$

where $r_{i}$ and $x_{i}$ are device-generated random values, s is the master secret of the TA, and $H_{1}(\cdot )$ is a collision-resistant hash function. These relations ensure that neither the TA nor the device alone can reconstruct the full private key, thereby eliminating certificate-based escrow.

#### Appendix B.1.2. Dynamic Membership and Rekeying

When a participant joins or leaves, the shared group key is refreshed using a CRT-based mechanism. Each device is assigned a unique modulus $b_{i}$ such that $gcd(b_{i},b_{j})=1$ for i≠j. The updated group-key component satisfies

(A3) $$\begin{array}{cccc} & a^{\prime }\;mod\;b_{i}=k_{d_{i}}^{\prime },i\neq r & & \end{array}$$

where r denotes the revoked node. This guarantees that only active members can reconstruct the new key, providing forward and backward secrecy during membership updates.

### Appendix B.2. Local Training and Authentication

This appendix lists the supplementary equations referenced in Section 3.1.2.

#### Signature Generation

Each device $w_{i}$ selects a random scalar $u_{i}\in Z_{q}^{*}$ and computes

(A4) $$U_{i}=u_{i}P$$

It then derives an authentication coefficient

(A5) $$a_{i}=H_{1}\;({PID}_{i},\;T_{i})$$

and forms the signature,

(A6) $${sgn}_{i}=u_{i}+h_{i2}*{SK}_{i}+S_{i}(mod\;q)$$

where $h_{i2}=H_{3}(W_{i}^{t},PID_{i},PK_{i},U_{i},T_{i})$, $SK_{i}$ is the device’s private key, and $S_{i}$ represents the broadcast group-key contribution. These equations collectively define the certificateless signing process used to authenticate local model updates.

### Appendix B.3. Edge-Side Verification and Screening

This appendix lists supporting relations referenced in Section 3.1.3.

#### Appendix B.3.1. Batch Verification

When multiple signed updates are processed concurrently, the edge applies batch verification to reduce computation:

(A7) $$\sum_{i=1}^{m}{sgn}_{i}P=\;\sum_{i=1}^{m}\left[U_{i}+h_{i2}\left(L_{i}+h_{i1}P_{pub}\right)+a_{i}K_{pub}\right]$$

#### Appendix B.3.2. Norm- and Loss-Based Screening

Each authenticated update’s scale and contribution quality are checked via

(A8) $$g_{i}=\left|\left|vec\left(W_{i}^{t}\right)\right|\right|{\left|\left|vec\left({\bar{W}}^{t}\right)\right|\right|}_{2},\;\Delta L_{i}=L\left({\bar{W}}^{t}\right)-L(W_{i}^{t})$$

to ensure stability and fairness before aggregation.

### Appendix B.4. Blockchain-Based Audit and Membership Management

This appendix summarizes supporting relations for Section 3.1.4.

#### Appendix B.4.1. Blockchain Voting and Validation

Each edge node $E_{j}$ casts a binary vote $validate_{j}(\Delta W_{i})$ for candidate update $W_{i}^{t}$; the total vote count is

(A9) $$V_{i}=\sum_{j=1}^{n}{validate}_{j}(\Delta W_{i})$$

and the update is accepted if a majority of edges approve $V_{i}\geq \frac{n}{2}$.

#### Appendix B.4.2. CRT-Based Rekeying

Each participant $w_{i}$ maintains a modulus $b_{i}$ and CRT coefficient $c_{i}=C/b_{i}$ satisfying $c_{i}e_{i}\equiv 1(modb_{i})$. The group parameter and key updates are computed as

(A10) $$\mu =\sum_{i=1}^{m}\mu_{i},\;\alpha =k_{d}*\mu$$

and for revocation of a member r, the adjusted values are,

(A11) $$\mu^{\prime }=\mu -\mu_{r},\;\alpha^{\prime }=k_{d}^{\prime }*\mu^{\prime }\;$$

These relations ensure that only active members can derive the new group key, maintaining forward and backward secrecy across membership changes.

### Appendix B.5. Security Model and Assumptions

This appendix summarizes the formal assumptions supporting Section 3.1.5.

1. Elliptic Curve Discrete Logarithm Problem (ECDLP): Given P and Q=sP on an elliptic curve $E/F_{q}$, finding s is computationally infeasible.
2. **Probabilistic Polynomial-Time (PPT) Adversary:** A operates within polynomial time, may issue chosen-message queries, and compromise up to m clients.
3. Existential Unforgeability (EUF-CMA): No PPT adversary can produce a valid ECC signature without the private key.
4. CRT-based Rekeying Assumption: Revocation triggers a rekeying event; only active clients receive the new parameters, ensuring forward/backward secrecy.
5. Blockchain Integrity Assumption: Consensus ensures committed records cannot be altered without detection.

These assumptions collectively underpin the four lemmas outlined above.

### Appendix B.6. Global Aggregation and Checkpointing

This appendix summarizes the supporting relations for Section 3.1.6.

#### Appendix B.6.1. Batch Verification

For a batch of m authenticated updates with signatures $\left\{sgn_{i}\right\}$ and random coefficients $\left\{\delta_{i}\right\}$, the small-exponent verification method evaluates:

(A12) $$\left(\sum_{i=1}^{m}\delta_{i}*{sgn}_{i}\right)*P={\sum_{i=1}^{m}\delta_{i}*U}_{i}+\sum_{i=1}^{m}\delta_{i}*h_{i2}\left(L_{i}+h_{i1}*P_{pub}\right)+\sum_{i=1}^{m}\delta_{i}$$

to validate all signatures simultaneously, where each $\delta_{i}$ binds the signatures into one verification equation.

#### Appendix B.6.2. Checkpoint Consistency

To ensure model-state continuity, each checkpoint $C_{t}$ is cryptographically linked to its predecessor via a block reference hash $H(C_{t}||C_{t-1})$, preventing rollback manipulation and ensuring verifiable continuity of the training timeline.

### Appendix B.7. Self-Healing Response

This appendix provides the supporting relation referenced in Section 3.1.7.

The self-healing function R(t) selects the appropriate corrective action as,

(A13) $$R\left(t\right)=\{\frac{W_{g}^{t-1},if\;rollback\;to\;the\;previous\;trusted\;checkpoint\;is\;required}{Reconfigure\left(W_{g}^{t}\right),if\;adaptive\;exclusion\;and\;reaggreagtion\;is\;applied}$$

This mechanism guarantees that the global model either reverts to the most recent verified state or adapts dynamically to isolate malicious contributions, ensuring continuity, transparency, and fault tolerance.

### Appendix B.8. Self-Healing Response

This appendix summarizes the supporting verification and recovery relations referenced in Section 3.1.8.

#### Appendix B.8.1. Acknowledgment Verification

Each device $w_{i}$ returns an acknowledgment digest of the received model:

(A14) $$H_{i}^{ack}=H({\hat{W}}_{g}^{t})\;$$

The edge verifies that all participating devices report the same digest before recording the acknowledgment set $\left\{PID_{i},H_{i}^{ack},T_{i}\right\}$ on the blockchain.

#### Appendix B.8.2. Recovery Verification

For devices reconnecting late, the stored blockchain record $B_{t}$ for round t is used to validate consistency:

(A15) H(W^gt)?=hm∈Bt

This ensures that delayed devices synchronize with the same authenticated global model as active participants.
